# Different Exosomal microRNA Profile in Aquaporin-4 Antibody Positive Neuromyelitis Optica Spectrum Disorders

**DOI:** 10.3389/fimmu.2020.01064

**Published:** 2020-05-29

**Authors:** Chen Chen, Yunting Wu, Miaochang Li, Chunping Cui, Yipeng Zhao, Xiaobo Sun, Yuge Wang, Chunxin Liu, Haotian Wu, Xiaonan Zhong, Allan G. Kermode, Lisheng Peng, Wei Qiu

**Affiliations:** ^1^Department of Neurology, The Third Affiliated Hospital of Sun Yat-sen University, Guangzhou, China; ^2^Department of Neurology, Zhaoqing No. 1 People's Hospital, Zhaoqing, China; ^3^Centre for Neuromuscular and Neurological Disorders, University of Western Australia, Perth, WA, Australia; ^4^Department of Neurology, Sir Charles Gairdner Hospital, Queen Elizabeth II Medical Centre, Perth, WA, Australia; ^5^Institute of Immunology and Infectious Diseases, Murdoch University, Perth, WA, Australia

**Keywords:** exosome, microRNAs, neuromyelitis optica spectrum disorders, multiple sclerosis, biomarker

## Abstract

Neuromyelitis optica spectrum disorders (NMOSD) and multiple sclerosis (MS) are inflammatory demyelinating diseases of the central nervous system. Exosomal microRNAs (miRNAs) are emerging biomarkers for demyelinating diseases. In this study, 52 aquaporin-4 antibody serum-positive NMOSD patients, 18 relapsing-remitting multiple sclerosis (RRMS) patients and 17 healthy controls (HCs) were included for the next-generation sequencing (NGS). To validate the NGS results, the valuable miRNAs were selected for validation by real-time quantitative polymerase chain reaction in another cohort of patients, comprising 31 NMOSD patients and 14 HCs. In addition, these miRNAs were also validated in a longitudinal study. NGS data revealed the exosomal miRNAs profile in NMOSD patients was different from HCs. Among those potential exosomal miRNAs which can distinguish NMOSD status, hsa-miR-122-3p and hsa-miR-200a-5p were the most abundant miRNAs. In addition, hsa-miR-122-3p and hsa-miR-200a-5p were significantly upregulated in the serum exosome of relapsing NMOSD compared with that in remitting NMOSD. Hsa-miR-122-3p and hsa-miR-200a-5p had positive correlations with disease severity in NMOSD patients. Kyoto Encyclopedia of Genes and Genomes pathway analysis revealed that the MAPK, Wnt and Ras signaling pathways were enriched. Further biological function analysis demonstrated that these two miRNAs might be involved in the immunoregulation of NMOSD pathogenesis. Our results indicated that miRNAs delivered by exosomes could be applied as potential biomarkers for NMOSD.

## Introduction

Neuromyelitis optica spectrum disorders (NMOSD) and multiple sclerosis (MS) are inflammatory demyelinating diseases in the central nervous system (CNS) with different pathogeneses. Immunoglobulin G antibody against aquaporin-4 (AQP4-IgG) is considered a highly specific biomarker and is present in more than 80% of NMOSD patients (1). AQP4-IgG targets aquaporin-4, inducing astrocytic damage by antibody-dependent cellular cytotoxicity mechanisms (2), and inducing the non-AQP4-expressing cells damage by antibody-dependent cellular cytotoxicity bystander killing mechanism (3). Although AQP4-IgG is widely recognized as the pathogenic factor of NMOSD, other mechanisms that may be involved are still worth studying.

microRNAs (miRNAs) are a class of short non-coding RNAs that are ~18 to 25 nucleotides in length. miRNAs perform vital biological functions and can be novel diagnostic and prognostic biomarkers in clinical practice. A recent study using next-generation sequencing (NGS) demonstrated that circulating miRNAs in NMOSD may permit its discrimination from MS (4). Exosomes are 30–120 nm membraned nanovesicles that are formed in the cytoplasm and secreted by virtually all cell types. Exosomes differ based on the cell of origin and are relatively easily detected in serum and other biological fluids (5). A recent study has demonstrated that exosomal protein spectrum changed after the plasma exchange in NMOSD patients and a series of complement components possibly associates with the pathogenesis of NMOSD (6). The biological functions of exosomes also rely on their cargos, such as miRNAs, long non-coding RNAs. miRNAs delivered by exosomes has already been found to play important roles in intercellular communication (7). A recent study demonstrated that relapsing-remitting MS (RRMS) patients exhibited altered exosomal miRNAs profile (8). However, serum exosomal miRNAs profiles have not yet been investigated in NMOSD, and the potential role in differentiating NMOSD from MS has not yet been explored.

## Materials and Methods

### Subjects

This study consecutively recruited 52 AQP4-IgG-positive NMOSD patients (17 in acute phase and 35 in remission), 18 RRMS patients (6 in acute phase and 12 in remission) and 17 healthy controls (HCs) for the NGS study, and another independent group of 31 NMOSD patients (16 in the acute phase and 15 in remission) and 14 HCs for the real-time quantitative polymerase chain reaction (RT-qPCR) validation of potential miRNAs. In addition, a longitudinal study was conducted to validate the different expressions of valuable miRNAs between acute phase and remission in the same patients (7 AQP4-IgG-positive NMOSD patients) by RT-qPCR. All the participants were recruited at the Third Affiliated Hospital of Sun Yat-Sen University from March 2017 to September 2018. To reduce inhomogeneity of NMOSD group, only AQP4-IgG positive NMOSD patients were included. All the NMOSD and MS patients meet the international panel diagnosis criteria for NMOSD and MS (9, 10). MS patients also meet the typical Barkhoff MRI criteria, and patients with atypical MRI features were excluded (11). HCs were selected after clinical examination and none of them had nervous system diseases. The clinical relapse was defined as an acute appearance of new symptoms lasting for at least 24 h, with new T2 lesions or enhanced T1 lesions on magnetic resonance imaging (MRI) and an increase in the expanded disability status scale (EDSS) score of over 1.0. The remission phase was defined as a period when the neurological condition of the patient had been stable for more than one month.

This study was approved by the ethics committee of the Third Affiliated Hospital of Sun Yat-Sen University. Informed written consent was obtained from all participants.

### Sample Collection and Antibody Testing

Blood were harvested from all participants after fasting for at least 4 h. All NMOSD patients were AQP4-IgG positive, while MS patients were negative. In addition, all participants were myelin oligodendrocyte glycoprotein (MOG) antibody negative. Blood was collected from patients in the acute stage before clinical treatment. Blood samples were stored at 4°C for 4 h to allow clotting. Then, samples were centrifuged at 2500 × g for 10 min at 4°C. Isolated serum (~4 milliliters) was preserved at −80°C for further analysis.

Serum immunoglobulin G antibody against MOG was tested by an in-house cell-based assay using live cells transfected with full-length human MOG. AQP4-IgG was detected using an indirect immunofluorescence assay (EUROIMMUN Medizinische Labor diagnostika AG, Germany).

### Exosome Isolation, Identification and RNA Extraction

A total of 3-4 mL of serum was mixed with Ribo™ Exosome Isolation Reagent (RiboBio, China) for exosome extraction. The presence and purity of exosomes were assessed by nanoparticle tracking analysis (NTA) and transmission electron microscopy (TEM). To determine the exosome concentration, the exosomal marker proteins CD63 and CD81 were labeled with monoclonal antibodies (BD Biosciences, USA) and analyzed by a BD Accuri C6 flow cytometer (BD Biosciences, USA). The exosome size was assessed by a ZETASIZER Nano SeriesNano-ZS (Malvern Instruments, Ltd., UK). Exosomes were processed for visualization by TECNAI 12 TEM System (Philips, Netherlands). Total exosomal RNA was extracted using a HiPure Serum/Plasma miRNA Kit (Magen, China) according to the manufacturer's protocol. After isolation, RNA quantification and purity were assessed by a Qubit 2.0 (Life Technologies, USA) and an Agilent 2200 TapeStation (Agilent Technologies, USA).

### Library Construction and Next-Generation Sequencing

Approximately 100 ng of total RNA per sample was used to construct a library using an NEBNext® Multiplex Small RNA Library Prep Set for Illumina® (Illumina, USA) and only small RNAs less than 30 nt were used for library preparation. RNA was qualified and was then amplified and sequenced using a HiSeq Rapid SBS Kit V2 (50 cycles) and a HiSeq Rapid SR Cluster Kit V2 on a HiSeq^TM^ 2500 platform (Illumina, USA).

### Validating by RT-qPCR

To validate the NGS results, we selected potential miRNAs for further analysis by RT-qPCR in another cohort of participants. The selected miRNAs should have different expressions between the acute phase and remission phase. Quantification of miRNAs expression was performed using a miRNA qPCR Starter Kit (RiboBio, China) on a C1000 Touch^TM^ Sequence Detection System (Bio-Rad, USA) according to the manufacturer's protocols. We chose the cel-miR-39-3p mimic, which has a stable expression between cells and exosomes, to normalize technical variation between the samples.

### Target Prediction and Functional Analysis

In this study, we used the miRDeep algorithm to match raw NGS reads to miRNAs. Subsequent calculations were performed by using the R statistical programming environment (version 3.0.2). To increase the comparability of the raw read counts from NGS, standard quantile normalization was used as implemented in the normalize-counts per million functions. We used four widely accepted databases to predict the mutual potential target genes of the candidate miRNAs: TargetScan (http://www.targetscan.org/vert_72/), miRDB (http://mirdb.org/), DIANA tools microT-CDS (http://diana.imis.athena-innovation.gr/DianaTools/) and miRDIP (http://ophid.utoronto.ca/mirDIP/). The target genes predicted by all four databases were selected for further biological function analysis and Kyoto Encyclopedia of Genes and Genomes (KEGG) pathway analysis by the Database for Annotation, Visualization and Integrated Discovery (DAVID) Bioinformatics Resources 6.8 (https://david.ncifcrf.gov/) and the Search Tool for Retrieval of Interacting Genes/Proteins (STRING) database v11.0 (https://string-db.org/).

### Statistical Analysis

The sex distribution among the groups was assessed by a 2 x 3 or 2 x 2 Chi-square test. Age differences between groups were assessed by analysis of variance. For clinical data, *p* values of < 0.05 were considered significant. The edgeR generalized linear model approach (version 3.22.5) was applied to determine differential expression between groups using log (counts per million) normalization. False discovery rate (FDR) adjustment was performed to account for multiple testing. To reduce the false positive rate, genes with an adjusted two-sided *P*-value of < 0.01 and a fold change in expression of >2 were considered differentially expressed.

## Results

### Participants

In the NGS study, a total of 52 NMOSD patients (17 in the acute phase and 35 in remission), 18 RRMS patients (6 in the acute phase and 12 in remission) were consecutively included. There were significant differences in the mean age and sex distributions among the three groups (*p* = 0.001 and *p* = 0.010, respectively), which was in accordance with the clinical characteristics (12). There's no difference in the proportion of patients who received immunotherapy between NMOSD and MS patients during this study (*p* = 0.188). The steroids dosage of patients included in the study was 8 mg methylprednisolone per day. There was no significant difference in the EDSS score between patients with NMOSD and MS, whereas patients in the acute stage scored higher than those in remission. Five MS patients were oligoclonal bands positive which was higher than NMOSD (*p* < 0.001). The clinical characteristics of participants in the NGS study were shown in Table 1.

**Table 1:** Clinical characteristics of the patients with NMOSD, RRMS and HCs in the NGS study.

	**NMOSD (*n* = 52)**	**RRMS (*n* = 18)**	**HCs (*n* = 17)**	***p***
Mean age at onset (range, y)	37.9(19–63)	26.8(14–53)	32.8(13–61)	0.001
Females/males (% female)	48/4(92.3)	12/6(66.7)	12/5(70.6)	0.010
Median disease duration (range, mo)	45.5(2–186)	33.5(3-100)	–	0.018
Median EDSS scores (range)	3.0(0–8.0)	2.5(0–7.5)	–	0.466
Positive rate of OCBs (%)	0	27.8(5/13)	0	<0.001
Immunotherapy (number)	AZA (13) AZA+S (4) MMF (4) MMF+S (16) S (6) None (9)	AZA (2) AZA+S (2) Tacrolimus (1) S (7) None (6)	–	–

*NMOSD, neuromyelitis optica spectrum disorders; RRMS, relapsing-remitting multiple sclerosis; HCs, healthy controls; NGS, next-generation sequencing; y, years; mo, month; EDSS, expanded disability status scale; OCBs, oligoclonal bands; AZA, azathioprine; MMF, mycophenolate mofetil; S, steroids*.

In the RT-qPCR validation study, a separate cohort of 31 NMOSD patients (16 in the acute phase and 15 in remission) and 14 HCs were included to validate the results of the NGS study. There was no significant difference in the sex distribution, mean age or disease duration among the groups. The median EDSS score of acute NMOSD patients was higher than that of remitting NMOSD patients. The clinical characteristics of 31 NMOSD patients and 14 HCs were shown in Table 2. In addition, serum samples during the acute and remitting stage were taken from each NMOSD patients (5 females and 2 males), in order to conduct a longitudinal study. The mean age at onset was 34.7 (22–57), median disease duration was 48 (18–155) months. The median EDSS scores was 5.0 (3-7) in the acute stage, which was higher than 2.0 (1-3) in the remission stage.

**Table 2:** Clinical characteristics of the 31 NMOSD patients and 14 HCs in the RT-qPCR validation study.

	**NMOSD in relapsing (*n* = 16)**	**NMOSD in remission (*n* = 15)**	**HCs (*n* = 14)**	***p***
Mean age at onset (range, y)	37.2(20–61)	38.6(21–60)	35.43(21–58)	0.751
Females/males (% female)	14/2(87.5)	13/2(86.7)	12/2(85.7)	0.990
Median disease duration (range, mo)	44(13–177)	47(24–134)	–	0.692
Median EDSS scores (range)	4.5(2.0–7.0)	2.0(0–3.0)	–	0.000
Immunotherapy (number)	AZA (2) AZA+S (2) MMF (2) MMF+S (5) S (1) None (3)	AZA (2) AZA+S (3) MMF (2) MMF+S (7) S (1)	–	–

*NMOSD, neuromyelitis optica spectrum disorders; HCs, healthy controls; RT-qPCR, realtime quantitative polymerase chain; y, years; mo, month; EDSS, expanded disability status scale; AZA, azathioprine; MMF, mycophenolate mofetil; S, steroids*.

### Exosome Isolation and Characterization

**Figure 1:**
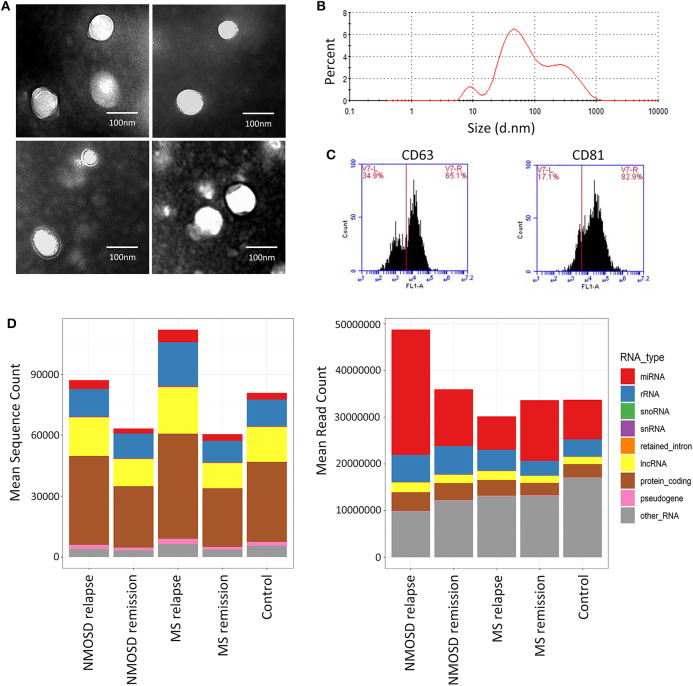
Analysis of serum exosomal miRNAs in NMOSD, MS and HCs by next-generation sequencing. **(A)** Transmission electron microscopy images of extracted serum exosome. The diameter of typical exosome is <100 nm. **(B)** The representative result of nanoparticle tracking analysis. **(C)** Two exosomal markers (CD63 and CD81) detected by flow cytometry. **(D)** The mean sequencing count and the mean read count per sample of different classes of RNA in NMOSD, MS, and HCs.

To assess the quality of exosomes, we randomly selected 8 samples for exosome assessment by TEM and NTA. TEM revealed typical rounded and membrane-bound particles with a homogeneous appearance (Figure 1). No morphological differences were observed between the particles from the three groups. In addition, NTA revealed that the size distribution of the exosomes ranged from 30 to 150 nm for all participants. Flow cytometry revealed that the average positive rates of CD63 and CD81 expression were 60.2 and 61.0%, respectively. These results indicated that the particles isolated from serum were qualified exosomes.

### Exosomal miRNAs Profile in Serum

**Figure 2:**
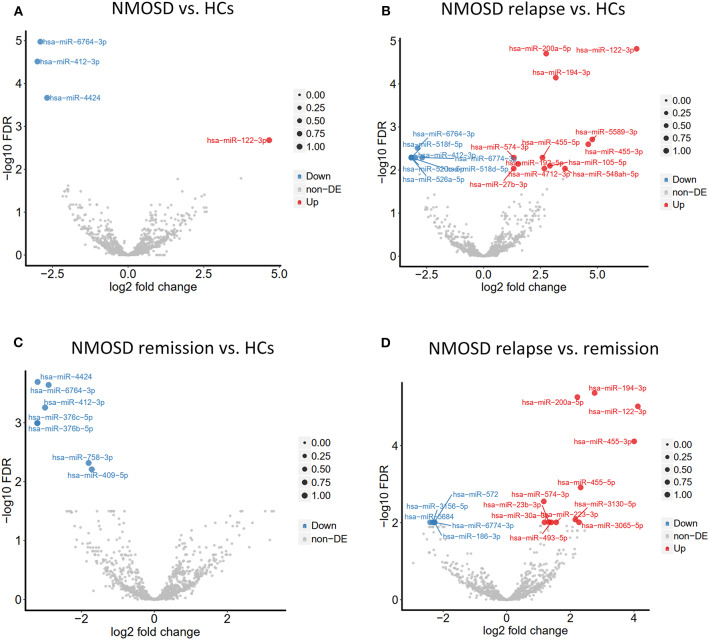
Volcano plots demonstrated different exosomal miRNAs profiles between NMOSD and HCs based on the NGS data. **(A)** The comparation of exosomal miRNAs profiles in NMOSD patients compared with HCs. **(B,C)** The comparations of exosomal miRNAs profiles between the subgroups of NMOSD and HCs. **(D)** The comparations of exosomal miRNAs profiles between relapsing NMOSD patients and remitting NMOSD patients. Non-DE, not differentially expressed.

To investigate the exosomal miRNAs profile in NMOSD, we carried out exosomal miRNAs sequencing. Interestingly, this study found different exosomal miRNAs profile in NMOSD (Table 3). Four miRNAs exhibited significantly altered expression in NMOSD compared with that in HCs. Hsa-miR-122-3p was upregulated and hsa-miR-4424, hsa-miR-6764-3p and hsa-miR-412-3p were downregulated in patients with NMOSD relative to their expression in HCs. Nineteen miRNAs were found differentially expressed in acute NMOSD patients compared with HCs. In the comparison of acute with remitting NMOSD, we found 17 significantly differentially expressed miRNAs. In addition, 7 miRNAs were downregulated in remitting NMOSD patients compared with HCs (Figure 2).

**Table 3:** Different exosomal miRNAs profile in NMOSD compared with HCs.

**NMOSD relapse vs. HCs**			**NMOSD remission vs. HCs**			**NMOSD relapse vs. remission**		
**miRNA**	**Log_**2**_FC**	**FDR**	**miRNA**	**logF_**2**_C**	**FDR**	**miRNA**	**Log_**2**_FC**	**FDR**
hsa-miR-122-3p	6.72	0.00002	hsa-miR-4424	−3.20	0.00020	hsa-miR-194-3p	2.76	0.00000
hsa-miR-5589-3p	4.78	0.00195	hsa-miR-6764-3p	−2.90	0.00023	hsa-miR-200a-5p	2.22	0.00001
hsa-miR-455-3p	4.60	0.00252	hsa-miR-412-3p	−3.00	0.00055	hsa-miR-122-3p	4.12	0.00001
hsa-miR-548ah-5p	3.57	0.00923	hsa-miR-376c-5p	−3.21	0.00102	hsa-miR-455-3p	4.00	0.00008
hsa-miR-194-3p	3.17	0.00007	hsa-miR-376b-5p	−3.21	0.00102	hsa-miR-455-5p	2.32	0.00122
hsa-miR-105-5p	2.91	0.00796	hsa-miR-758-3p	−1.80	0.00482	hsa-miR-574-3p	1.16	0.00281
hsa-miR-200a-5p	2.75	0.00002	hsa-miR-409-5p	−1.72	0.00620	hsa-miR-3130-5p	2.16	0.00829
hsa-miR-4712-3p	2.67	0.00923				hsa-miR-23b-3p	1.32	0.00965
hsa-miR-455-5p	2.59	0.00514				hsa-miR-3065-5p	2.27	0.00979
hsa-miR-192-5p	1.52	0.00721				hsa-miR-223-3p	1.55	0.00979
hsa-miR-574-3p	1.33	0.00514				hsa-miR-493-5p	1.40	0.00979
hsa-miR-27b-3p	1.32	0.00923				hsa-miR-30a-3p	1.19	0.00979
hsa-miR-6774-3p	−2.70	0.00514				hsa-miR-6774-3p	−2.27	0.00979
hsa-miR-6764-3p	−2.90	0.00307				hsa-miR-186-3p	−2.27	0.00979
hsa-miR-412-3p	−3.00	0.00514				hsa-miR-572	−2.28	0.00979
hsa-miR-518f-5p	−3.17	0.00514				hsa-miR-3156-5p	−2.32	0.00979
hsa-miR-526a-5p	−3.17	0.00514				hsa-miR-5684	−2.41	0.00979
hsa-miR-520c-5p	−3.17	0.00514						
hsa-miR-518d-5p	−3.17	0.00514						

Among the differentially expressed miRNAs, hsa-miR-122-3p and hsa-miR-200a-5p had an average of ~100 reads in patients in the acute phase. In addition, hsa-miR-122-3p and hsa-miR-200a-5p were upregulated in acute NMOSD relative to their expression in patients with remitting NMOSD and HCs, with > 2-fold changes and significant differences.

This study also enrolled 6 acute and 12 remitting MS patients for comparison with NMOSD patients and HCs. However, no miRNA was found to be differentially expressed between MS group and NMOSD group or HCs. No difference was found between the acute MS and remitting MS or HCs. When we compared remitting MS with HCs, we found that hsa-miR-582-3p and hsa-miR-542-3p were upregulated in remitting MS patients. Finally, we compared the exosomal miRNA expression profile of NMOSD with that of MS. We found that miR-320a-5p was significantly downregulated in acute NMOSD relative to its expression in acute MS. Six miRNAs were also downregulated in remitting NMOSD relative to their expression in remitting MS, that were hsa-miR-380-3p, hsa-miR-3145-3p, hsa-miR-216a-5p, hsa-miR-548ba, hsa-miR-153-3p, hsa-miR-448 (Table 4).

**Table 4:** Different exosomal miRNAs expressions in MS compared with NMOSD and HCs.

	**miRNA**	**Log_**2**_FC**	**FDR**
MS remission vs. HCs	hsa-miR-582-3p	2.68	0.00248
	hsa-miR-542-3p	2.42	0.00248
NMOSD relapse vs. MS relapse	hsa-miR-320a-5p	−4.16	0.00020
NMOSD remission vs. MS remission	hsa-miR-380-3p	−3.54	0.00059
	hsa-miR-216a-5p	−3.19	0.00278
	hsa-miR-3145-3p	−2.72	0.00278
	hsa-miR-548ba	−2.55	0.00278
	hsa-miR-153-3p	−2.76	0.00308
	hsa-miR-448	−3.03	0.00376

Based on the results shown above, we found evidence that exosomal miRNAs were differentially expressed between NMOSD and MS or HCs. Hsa-miR-122-3p and hsa-miR-200a-5p were most abundant and valuable in distinguishing NMOSD relapse.

### RT-qPCR Validation

In this part of study, hsa-miR-122-3p and hsa-miR-200a-5p were screened as candidates for further validation. We did not recruit MS patients in this part of the study because of the limited findings in the NGS study of MS. Hsa-miR-194-3p and hsa-miR-455-3p were not selected for validation due to the low expression in the serum exosome.

**Figure 3:**
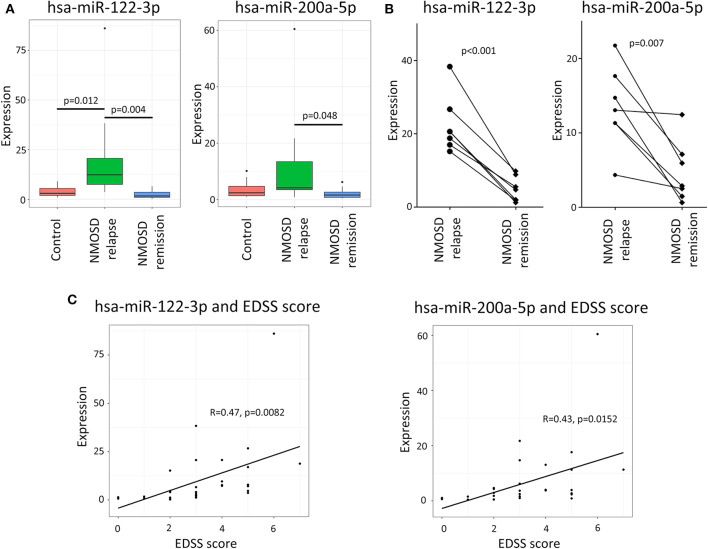
The validation of potential exosomal miRNAs by RT-qPCR and the correlation of miRNAs with EDSS scores. **(A)** The RT-qPCR validation demonstrated that the expressions of hsa-miRNA-122-3p and hsa-miRNA-200a-5p were significantly higher in relapsing NMOSD than in remission. **(B)** The longitudinal study demonstrated that the expressions of hsa-miRNA-122-3p and hsa-miRNA-200a-5p were higher in relapsing than in remitting NMOSD patients. **(C)** The analysis showed that the expressions of hsa-miR-122-3p and hsa-miR-200a-5p were positively correlated with EDSS scores of NMOSD patients based on the RT-qPCR data.

The RT-qPCR results revealed the same trend as the NGS data. According to the expression of hsa-miR-122-3p was significantly higher in relapsing than in remitting NMOSD or HCs (*p* = 0.004 and *p* = 0.012, respectively). The mean expression level of hsa-miR-122-3p was higher in HCs than in remitting NMOSD patients, but the difference was not significant (*p* = 0.058). Hsa-miR-200a-5p was also significantly overexpressed in relapsing NMOSD compared with remitting NMOSD (*p* = 0.023). However, no significant differences were found between NMOSD subgroups and HCs (Figure 3A).

In addition, the longitudinal study demonstrated that the expressions of hsa-miRNA-122-3p and hsa-miRNA-200a-5p were higher in relapsing than in remitting NMOSD patients (*p* < 0.001 and *p* = 0.007, respectively) (Figure 3B).

### Correlations Between miRNAs Expression and Clinical Characteristics

Hsa-miR-122-3p and hsa-miR-200a-5p were selected to analyse the correlation between miRNAs and clinical characteristics. Interestingly, we found that the expressions of hsa-miR-122-3p and hsa-miR-200a-5p had positive correlations with EDSS scores for all NMOSD patients (*R* = 0.47, *p* = 0.0082 and *R* = 0.43, *p* = 0.0152) according to the RT-qPCR data (Figure 3C). The correlation of hsa-miR-122-3p expression with EDSS score was also proved by the NSG data (R = 0.40, *p* = 0.0029). However, no correlation of hsa-miR-200a-5p was found according to the NGS study (R = 0.24, *p* = 0.089). No correlations between EDSS scores and the expressions of hsa-miRNA-122-3p and hsa-miRNA-200a-5p in relapsing NMOSD patients were found based on the RT-qPCR data (R = 0.175, *p* = 0.517, and R = 0.234, *p* = 0.384, respectively). The expressions of hsa-miRNA-122-3p and hsa-miRNA-200a-5p were not significantly different between NMOSD patients with attacks in the spinal cord and in the optic nerves, based on the NGS study (*p* = 0.158 and *p* = 0.279, respectively) and the RT-qPCR study (*p* = 0.231 and *p* = 0.392, respectively). No correlation was found between the selected miRNAs and age, gender, disease course, number of attacks or oligoclonal bands status. In addition, no significant difference was found between NMOSD patients with or without receiving steroids according to the NGS data (*p* = 0.752 and *p* = 0.129, respectively) or the RT-qPCR data (*p* = 0.633 and *p* = 0.252, respectively).

### Gene Target Prediction and Pathway Analysis

**Figure 4:**
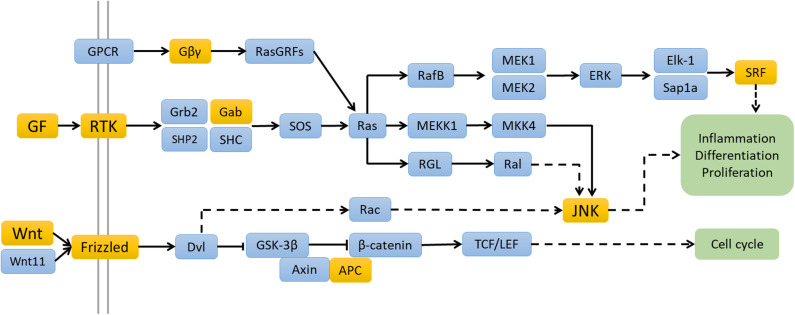
The possible signaling pathways of hsa-miRNA-122-3p and hsa-miRNA-200a-5p enriched by the KEGG pathway analyses. The genes with yellow backgrounds are the target genes of hsa-miRNA-122-3p and hsa-miRNA-200a-5p.

These four miRNAs databases (13–16) revealed 22 overlapping target genes for hsa-miR-122-3p and 365 overlapping target genes for hsa-miR-200a-5p. KEGG pathway analysis of hsa-miR-122-3p by DAVID and STRING both indicated enrichment of mitogen-activated protein kinase (MAPK) signaling pathway, with four genes involved. Regarding hsa-miR-200a-5p, KEGG pathway analysis revealed 5 enriched pathways, including regulating pluripotency of stem cells (*p* = 0.013), pathways in cancer (*p* = 0.020), Ras signaling pathway (*p* = 0.038), and Wnt signaling pathway (*p* = 0.043) (Figure 4). However, no KEGG pathway was enriched by STRING database.

## Discussion

In this study, we report a different serum exosomal miRNAs profile in patients with NMOSD compared with that in MS or HCs. During the identification of exosomal RNA, we identified 34 different classes of RNA, including miRNA, ribosomal RNA, small nucleolar RNA, long non-coding RNA, retained introns, small nuclear RNA, mitochondrial transfer RNA, pseudogenes and other RNAs. The key findings of this study were as follows: (i) the NGS RNA-sequence data revealed an miRNA pattern in patients with NMOSD different from that in HCs; (ii) hsa-miR-122-3p and hsa-miR-200a-5p were significantly upregulated in acute NMOSD relative to their levels in remission, the expressions were age- and sex-independent (17, 18); (iii) hsa-miR-122-3p had a positive correlation with the EDSS score in NMOSD and MS; and (iv) the MAPK, Wnt and Ras signaling pathways, which might participate in immunoregulation in NMOSD pathogenesis, were found to be enriched in the biological function analysis.

MiR-122-3p is a product of miR-122, serum exosomal hsa-miR-122-3p was found to be upregulated in chronic atrophic gastritis and could be a promising diagnostic biomarker (19). MiR-122-3p expression was also found to be decreased in the plasma of patients with rheumatoid arthritis (20). A study on MS demonstrated that the level of serum exosomal hsa-miR-122-3p was significantly decreased in relapsing MS patients compared with that in patients in remission and controls (8). In this study, our data demonstrated that the level of exosomal hsa-miR-122-3p was increased in the serum of acute NMOSD patients compared with that in remitting patients and HCs. Exosomal hsa-miR-122-3p expression correlated with the EDSS score, indicating that serum exosomal hsa-miR-122-3p might indicate NMOSD relapse.

MiR-200a-5p is one of the products of miR-200a, which is involved in various autoimmune diseases. Hsa-miR-200a expression was higher in CD4+ T cells from patients with relapsing MS than in those of remitting MS and HCs. Molecular signaling pathway enrichment analysis revealed that miR-200a might induce T helper 17 cell differentiation via the Janus kinase-signal transducer and activator of transcription, transforming growth factor-β and mammalian target of rapamycin pathways (21). A study revealed that hsa-miR-200a expression was significantly decreased in patients with rituximab-treated NMO, with a fold change of 0.21 relative to that in untreated patients (22). However, no expression difference of hsa-miR-122-3p and hsa-miR-200a-5p in the serum and whole blood was found between NMOSD and HCs (4). In this study, we found that hsa-miR-200a-5p expression was significantly elevated in patients with acute NMOSD compared with that in NMOSD in remission.

To better understand the consequences of hsa-miR-122-3p and hsa-miR-200a-5p dysregulation, we searched their target genes and analyzed their potential biological functions. The functional annotation revealed that the targets of hsa-miR-122-3p were related to the immunoglobulin domain terms. Four target genes of hsa-miR-122-3p were involved in the MAPK signaling pathway. The MAPK signaling pathway is widely involved in disorders of the innate and adaptive immune systems (23). This study revealed that the Ras signaling pathway and Wnt signaling pathway were enriched based on the target genes of hsa-miR-200a-5p. The Ras signaling pathway has multiple modulatory effects on cell proliferation and inflammatory responses. Altered Ras expression in T lymphocytes can result in autoimmune disorders such as systemic lupus erythematosus (24).

Several limitations of our study should be noted. Firstly, the number of RRMS patients included in the NGS study was small, we didn't find valuable dysregulated miRNAs in RRMS patients compared with HCs. This result might also be caused by different disease-modifying therapies in RRMS patients. Secondly, this study did not conduct research on the precise origin and potential target of the exosome and miRNAs. Therefore, it is difficult to define the specific destinations and biological function of the exosomal miRNAs. Further study on larger samples should be performed to validate our results, and focused on the precise mechanisms of exosomes and dysregulated exosomal miRNAs.

## Conclusion

In summary, our study demonstrated that profile of miRNAs delivered by exosomes in NMOSD with seropositive AQP4-IgG was different from that in MS or HCs. Exosomal microRNAs could be applied as potential biomarkers for NMOSD.

## Data Availability Statement

The raw data supporting the conclusions of this article will be made available by the authors, without undue reservation, to any qualified researcher.

## Ethics Statement

This study was approved by the ethics committee of the Third Affiliated Hospital of Sun Yat-Sen University. Informed written consent was obtained from all participants.

## Author Contributions

WQ, LP, and CCh determined the study design and performed the study. CCh was responsible for data collection and analyzed the data. CCh and WQ drafted the manuscript. YWu, ML, CCu, XS, YZ, YWa, CL, HW, XZ, and AK participated in data analysis and figure illustration. All authors read, critically revised, and approved the final manuscript.

## Conflict of Interest

The authors declare that the research was conducted in the absence of any commercial or financial relationships that could be construed as a potential conflict of interest.
